# Comparison of the Effects of Low Volume Prilocaine and Alkalinized Prilocaine for the Regional Intravenous Anesthesia Technique in Hand and Wrist Surgery

**DOI:** 10.1155/2014/725893

**Published:** 2014-07-15

**Authors:** Ozlem Kapusuz, Guldeniz Argun, Murat Arikan, Guray Toğral, Aysun Basarir, Nihal Kadiogullari

**Affiliations:** ^1^Department of Anesthesiology and Reanimation, Training and Research Hospital, Vatan Caddesi No. 33, Demetevler, 06200 Ankara, Turkey; ^2^Department of Orthopedics and Traumatology, Oncology Training and Research Hospital, Vatan Caddesi No. 33, Demetevler, 06200 Ankara, Turkey

## Abstract

*Aim*. Comparing the effectivity of prilocaine and prilocaine alkalinized with 8.4% NaHCO_3_ in terms of sensory and motor block onset and termination durations in RIVA technique considering patients' satisfaction and tolerance with application of tourniquet undergoing hand-wrist surgery.* Materials and Methods*. 64 patients were randomised into two groups. First group (Group P) was administered prilocaine and second group (Group PN) was administered prilocaine + %8.4 NaHCO_3_. Sensory and motor block onset and termination times and onset of tourniquet pain were recorded. *Results*. No significant difference was found between the two groups in terms of onset and termination of sensory block and the onset of motor block. The duration of the motor block was longer in Group PN than in Group P (*P* < 0.05). Tourniquet pain was more intense in Group P (*P* = 0.036). In Group PN, the use of additional drugs was recorded at a lower rate and patients' satisfaction was higher than Group P. * Conclusion*. In the present study, it was established that alkalinization of prilocaine had no effect on the duration of sensory block and it prolonged the duration of motor block, increased patients' satisfaction, and decreased tourniquet pain. It is our suggestion that future studies should be carried out on the issue by using different volumes.

## 1. Introductıon

Regional anesthesia applications gradually become more up-to-date and preferred methods. It is preferred to general anesthesia because of its advantages including leaving the airways open, preservation of airway reflexes, and reducing the risk of aspiration risk in emergency patients [1]. Regional intravenous anesthesia (RIVA) method is one of these methods.

RIVA is frequently preferred in patients particularly who will undergo operations on upper extremities since it provides easy application, effective anesthesia with rapid onset and termination of the effect, and short durations of the perioperative morbidity and postoperative hospital stay [2]. The RIVA technique has some disadvantages including high volume of local anesthetic agent and pain, related to tourniquet usage and short duration of anesthesia once the tourniquet is opened. Researches were carried out to reduce the side effects and improve the anesthetic quality and several adjuvant substances were added to local anesthetics. Such substances include opioid analgesics (morphine, meperidine, fentanyl, sufentanil), antihistaminics, muscle relaxants (atracurium), alpha-2 mimetics (clonidine, dexmedetomidine), nonsteroid anti-inflammatory agents (ketorolac, tenoxicam, acetyl salicylate, paracetamol), ketamine, and magnesium combinations which are thought to potentiate the effects of local anesthetics [3–7]. Alkalization of local anesthetics has also been used in regional anesthetic applications with the purpose of shortening the onset of local anesthetics and elongating the anesthetic effect [8–11].

The cytoplasm of the nerve fiber is more acidic than the extracellular fluid (pH = 6.9) and, therefore, ionization rates and consequently effectiveness of the drug molecules entering the cytoplasm will increase. Accordingly, the nonionized form helps the effectiveness of the efficacy of the drug by ensuring reaching the drug in the targeted site of effect. Decreasing the environmental pH increases the ionization rates of local anesthetics. Solutions of local anesthetic salts in water used in medicine display acidic reactions. These must be neutralized by the tissue fluids to be effective. Adding alkaline substances to local anesthetic solutions increases the local anesthetic effect by increasing the ratio of the nonionized form and making the penetration to the nerve body easier [12].

We aimed to compare the onset and termination times of sensory and motor block, tourniquet tolerance, patient satisfaction, and intraoperative and postoperative hemodynamic adverse effects with prilocaine and alkalinized local anesthetic obtained by adding 8.4% NaHCO_3_ to prilocaine patients that will undergo hand or wrist operations.

## 2. Materials and Methods

After obtaining the approval of the ethical committee (T.R. Ministry of Health, General Directorate of Drugs and Pharmacy, Ethical Committee, September 30, 2010/Number B-10-0-IEG-0-15-00-01) and consents of patients, 64 adult patients aged between 18 and 65 in ASA I-III risk group, who were planned to have hand and wrist operations were included in the study. Cases in which RIVA application is contraindicated including allergy against prilocaine, thrombophlebitis and atherosclerotic vascular diseases, Reynaud disease, arteriovenous fistula, scleroderma, sickle cell anemia, wide burns in the operation area, presence of laceration and infection, bleeding disorders, noncooperating patients, debilitated patients, and patients with malnutrition, and cases who refused to be operated on with this technique were excluded from the study.

On the operation day, patients were taken to the operation room 45 minutes before the operation and were premedicated with intramuscular 0.07 mg/kg midazolam. I.V. access was established with 20 gauge angiocath on the dorsal side of the hand that operation was not planned, and crystalloid infusion was started. The upper extremity to be operated was fixed after checking the pulse and establishing the I.V. access with 22 gauge angiocath on the dorsal side.

Demographic data of the patients on the operation table were recorded. Systolic arterial pressure (SAP), diastolic arterial pressure (DAP) and the mean arterial pressure (MAP), heartbeat rate (HR), electrocardiograph (ECG), and peripheral oxygen saturation (SPO_2_) with pulse oximeter were monitored.

Keeping the arm in elevation with vertical angle for 3 minutes to ensure evacuation of the venous blood with gravity, Esmarch bandage was applied tightly from distal to proximal to complete the evacuation of blood. The proximal cuff of the double-cuff tourniquet was inflated to 100–150 mmHg over the systolic arterial pressure measured on the same arm or up to 260 mmHg, and establishment of the occlusive pressure was confirmed with the disappearance of the radial pulse.

Patients were randomly divided into 2 groups, and Group 1 (Group P) received prilocaine with regional intravenous anesthesia technique and Group 2 (Group PN) received prilocaine + NaHCO_3_ as follows.

 GROUP P (*n* = 32):  3 mg/kg prilocaine 2% was completed to 20 mL with NaCl 0.9%.
 GROUP PN (*n* = 32):  3 mg/kg prilocaine 2% + 8.4% NaHCO_3_ (1 mec for every 10 mL, so 2 mec NaHCO_3_) was completed to 20 mL with NaCl 0.9%.

Immediately after the inflation of the proximal tourniquet, the solution prepared for each group was injected by the anesthesiologist through the cannula on the dorsum of the hand that operation was planned for within 90 seconds. The time for the establishment of sensory block after the injection was determined with pinprick test determined on six areas in the dermatomes of the median, radial, and ulnar nerves every 30 seconds. The period of establishment of the motor block was recorded as the time passed till the patient had become unable to move his/her fingers from the injection of the drug. This was compatible with Bromage 2 (Table 6). When the sensory block was established in all the dermatomes, the distal tourniquet was inflated to 260 mmHg pressure, the proximal tourniquet was opened, and operation was started.

Blood pressure, pulse, and saturation were followed before and immediately after the application of the tourniquet, every 5 minutes intraoperatively and every 10 minutes in the postoperative period. Presence of pain was questioned intraoperatively. Dormicum 0.01 mg/kg IV was applied to patients with VAS score was 2 to 4. Fentanyl 1 mcg/kg was applied to patients with VAS scores 4 to 6, and 0.01 mg/kg dormicum together with 1 mcg/kg fentanyl was applied to patients with VAS scores between 6 and 8 indicating severe pain. Motor block evaluation was carried out using the Bromage scale with scorings between 0 and 3 (Table 6). Patient satisfaction was classified according to the additional drugs administered. Classification was made using the numerical scale with scoring between 1 and 4. None of the patients required general anesthesia.

*Verbal Analog Scale (VAS) Was as Follows.*

 0–2: no pain,
 2–4: mild pain,
 4–6: medium-level pain,
 6–8: severe pain,
 8–10: very severe pain.

*The Numerical Scale Was Used for the Evaluation of Patient Satisfaction.*

 very good (1): patient is comfortable, and there is no need for analgesics or sedation,
 good (2): minor sedation is required,
 medium (3): patient needs additional analgesics,
 poor (4): patients that analgesia and sedation are administered together.

*Motor Block Level Was Evaluated with Bromage Scale.*

 0: no paralysis. Wrist, fingers, and forearm are able to move freely,
 1: wrist and/or elbow movements cannot stand resistance; finger movements are preserved,
 2: the patient lifts his/her hand from the wrist or forearm,
 3: there is no movement in the forearm.

It was planned that the tourniquet would be deflated not earlier than 30 minutes after the injection of the anesthetic drug and it would not stay inflated more than 2 hours. The period from the deflation of the tourniquet till the return of pain with pinprick test on the radial, median, and ulnar nerve dermatomes was determined as the reversal time of the sensory block, and the time passed till the patient is able to move his/her fingers was determined as the reversal time of the motor block. Vertigo, nausea and vomiting, double vision, gray-out, tinnitus, sleepiness, feeling of cold and chills, dizziness, and palpitation were questioned in the postoperative intensive care unit and adverse effects were followed up for 2 hours.

Blood pressure, pulses, and oxygen saturation of the patients were continued every 10 minutes for the patients in the postoperative intensive care unit. When the sensory block was terminated and pain started, intramuscular diclofenac sodium was administered to patients if they had VAS 4 or higher. Later, patients were transported to the ward with the recommendation of oral analgesics in case they have pain.

## 3. Size of Sampling and Power

G∗Power package program was used to determine the number of subjects to be included in the study. It was determined that at least 28 patients would be required in each group to determine the effect difference of *d* = 0.90 with 95% power between prilocaine (Group P) and prilocaine + NaHCO_3_ (Group PN) groups with *α* = 0.05 Type I and *β* = 0.05 Type II error rates. With the purpose of compensating the possible data loss and preventing the loss of power of the study, it was decided to add spare subjects to each group with a percentage of 15% (4 individuals for each group) and to start the study with 32 subjects in each group and at least 34 subjects in total.

### 3.1. Study Design

The study was planned as a randomized, controlled clinical trial. The sixty-four patients planned to be included in the study were randomly assigned into groups P and PN by taking their demographic characteristics into consideration using a special computer program (Random Alloc).

### 3.2. Statistical Analyzes

Compliance of the continuous variables obtained from patients with the normal distribution was evaluated with Shapiro-Wilk test. While the variables including age, BMI, dosage of local anesthetic, tourniquet time, and period of pain in hand did not comply with normal distribution, the onset time of the tourniquet pain and onset and termination times of sensory and motor blocks were consistent with the normal distribution. Numbers and percentages were used to indicate the descriptive statistics for the categorical variables (ASA score: presence of tourniquet pain, type of additional drugs: Bromage Scale). Distribution of the categorical variables (including gender and ASA scores) in P and PN groups were examined with cross tables, chi-square, and chi-square likelihood ratio tests. Mean ± standard deviation or median (IQR-Interquartile Range) was used to indicate the continuous variables depending on the normal distribution. Whether or not the continuous variables differed in groups P and PN was examined with t-test or with its nonparametric alternative, the Mann-Whitney test again based on the normal distribution.

MS-Excel 2003 and SPSS for Windows Ver. 15.0 (SPSS Inc, Chicago, IL., USA) programs were used for statistical analyses and calculations. For the statistical decisions, the *P* < 0.05 level was determined as the indicator of the significance of differences.

### 3.3. Findings

Nine of the patients (28.1%) were males in Group P evaluated in the frame of the study; the number of male patients in Group PN was 7 (21.9%). The gender distribution in Groups P and PN was statistically similar (*χ* ^2^ = 0.333; *P* = 0.564). Ages of patients ranged between 18 and 65 years of age. While the ages of our patients in Group P ranged between 18 and 62 years, the ages of our patients in Group PN ranged between 24 and 65 years. The mean ages of our patients in Groups P and PN were not statistically different (*Z* = 1.365; *P* = 0.172). The body weights of our patients ranged between 51 and 105 kg. The body weights in the Group P ranged between 50 and 105 kg while the same in the Group PN ranged between 51 and 104 kg (Table 1). The mean body weights of our patients in Groups P and PN were not statistically different (*Z* = 0.813; *P* = 0.416).

No statistical differences were found as regards median dosage of the local anesthetic between Groups P and PN (*Z* = 0.981; *P* = 0.327). The dosages of the local anesthetic were similar in Groups P and PN.

While the tourniquet time in Group P was minimum 30 minutes and maximum 54 minutes, the mean tourniquet time was 41 minutes (IQR = 8). In Group PN, however, the tourniquet time ranged between 30 and 75 minutes; the mean value was determined as 40 minutes (IQR = 12). No significant differences were found between Groups P and PN as regards tourniquet times (*Z* = 0.525; *P* = 0.600). The tourniquet application times were similar in the two groups.

**Figure 1:**
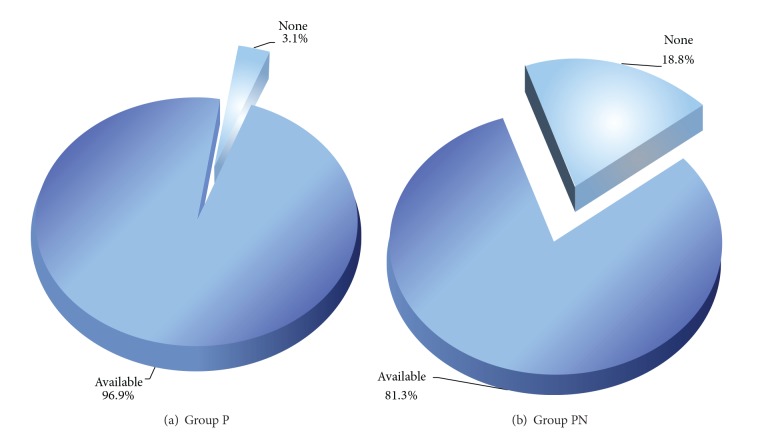
The rate of tourniquet pain in groups P and PN (*P* < 0.05).

While tourniquet pain was present in 31 patients in Group P (96.9%), the same rate was significantly lower in Group PN with 81.3% (*n* = 26) as compared to Group P (*χ* ^2^ = 4.402; *P* = 0.036). Less tourniquet pain was observed in Group PN as compared to Group P (Figure 1).

The tourniquet pain started at minimum 5 minutes and maximum 35 minutes, in Group P. The mean onset of the tourniquet pain was determined as 23.42 ± 3.88 minutes. These values were found as 4 and 45 minutes, respectively, and mean value as and 19.69 ± 12.02 in Group PN. We did not find any statistically significant differences between the Groups P and PN as regards the onset time of the tourniquet pain (*t* = 1.401; *P* = 0.169) (Table 2). The tourniquet pain in Groups P and PN appeared at statistically similar times.

**Figure 2:**
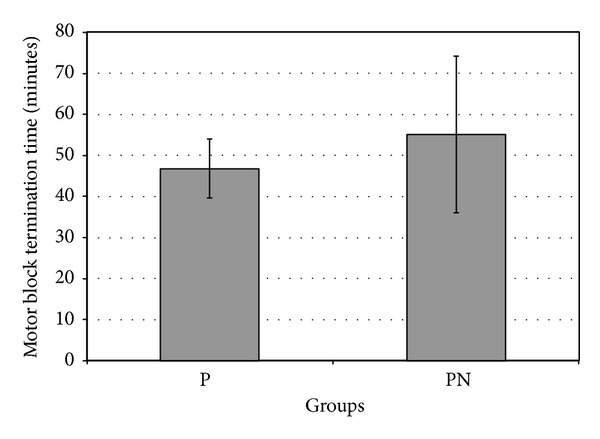
Mean values of motor block termination times in Groups P and PN.

Mean values of the onset and termination times of the sensory block did not differ significantly between Groups P and PN (*t* = 0.296; *P* = 0.769 and *t* = 0.401, *P* = 0.690, resp.). Also, the onset of the motor block (Bromage 2) was similar in Groups P and PN, and we did not find any significant differences (*t* = 0.062; *P* = 0.951) (Table 3). In the termination time of the motor block (Bromage 0), however, in patients in the Group PN, the motor block termination was later as compared to the patients in Group P (8.30 minutes), and this difference is significant statistically (*P* < 0.05) (*t* = 2.175; *P* = 0.034) (Figure 2).

Considering the times of appearance of pain at hand, it was seen that this time ranged between 45 and 130 minutes in Group P, and the median time for the appearance of pain in the hand was 72.50 (IQR = 23.75) minutes. The observation of pain at hand in Group PN ranged between 5 and 120 minutes (mean: 70.00 (IQR = 30.00) minutes). No significant differences were found between Groups P and PN regarding the times of appearance of pain in the hand (*Z* = 0.081; *P* = 0.936).

## 4. Requirement for Additional Drugs

No additional drug was used for patients with VAS scores between 0 and 2. Dormicum was added for the patients whose VAS scores could not be evaluated properly because of their agitation with the consideration that their VAS scores could be between 2 and 4 and their pain was questioned again. Fentanyl was administered to those with VAS scores between 4 and 6; fentanyl + dormicum combination was administered to those with VAS scores between 6 and 8. It was planned that general anesthesia would be shifted to for patients with VAS scores 8 or higher (Table 4). None of our patients had pain severe enough to require general anesthesia.

When the additional drugs used were examined, it was recorded that there was no need for additional drugs in 14 patients of Group P and in 9 patients in Group PN. While the rate of patients using Fentanyl was 2 (11.1%) in Group P, the same was 3 (13.0%) in Group PN. Dormicum and fentanyl + dormicum were used in 8 patients for each (44.4% each) in Group P, dormicum was used in 18 patients (78.3%) in Group PN, and fentanyl + dormicum was used in 2 (8.7%) patients.

While 10 patients (55.6%) who had used additional drugs in Group P had Fentanyl, 8 (44.4%) had dormicum. The same rates in Group PN were 5 (21.7%) and 18 (78.3%), respectively. The rate of Fentanyl use in Group P was significantly higher as compared to Group PN (*P* < 0.05) (*χ* ^2^ = 4.977; *P* = 0.026). The rate of dormicum in Group PN, however, was significantly higher as compared to Group P (*P* < 0.05) (Table 5).

Arm movements were evaluated with Bromage scale in both groups. In the evaluation, it was not possible to evaluate one patient in Group P, and Bromage scale was completed in 31 patients in Group P and 32 patients in group PN. Distribution of the Bromage scale according to groups is given in (Table 6). It is seen that the Bromage classification results in Groups P and PN are similar (*χ* ^2^ = 1.906; *P* = 0.59).

**Figure 3:**
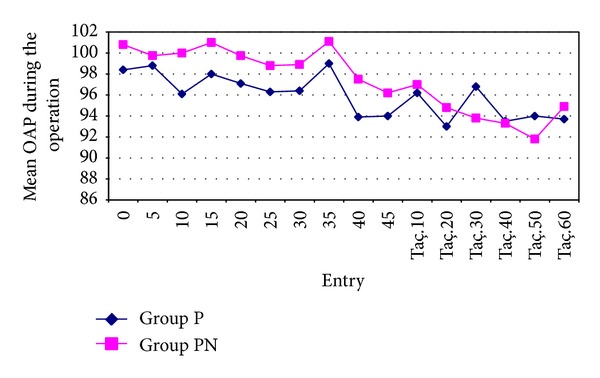
The mean OAP during the operation in Groups P and PN.

**Figure 4:**
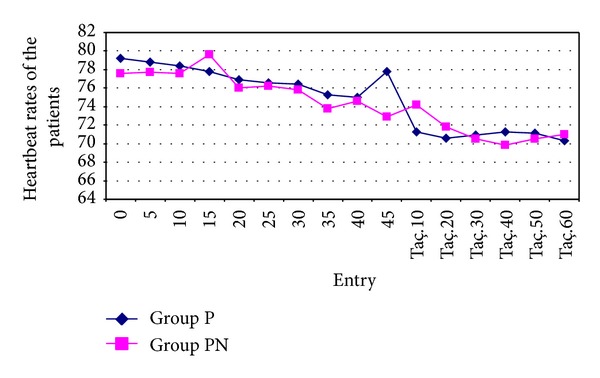
Heartbeat rates of the patients in Groups P and PN were similar.

The mean arterial pressure (MAP) values of the patients included in the study group were calculated with the help of systolic and diastolic arterial pressures recorded during the operation. MAP values and the heart rates of the patients in Groups P and PN were similar at all the measurement points. No significant differences were found between the preoperative MAP and postoperative MAP values in Groups P and PN (*P* > 0.05) (Figures 3 and 4).

Adverse effects including arrhythmia, vertigo, nausea, double vision, gray-out, tinnitus, dizziness, sleepiness, or tremor were seen in none of the patients in Groups P and PN. No statistically significant differences were found between the two groups (*P* > 0.05).

**Figure 5:**
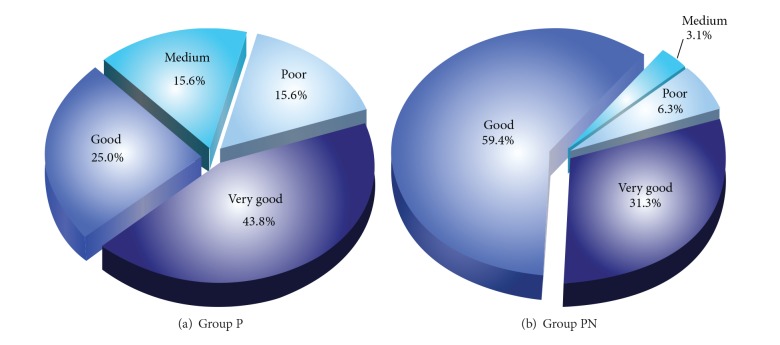
Patient satisfaction in Groups P and PN.

Patient satisfaction was examined in Groups P and PN. While the ratio of the patients in Group P that stated their satisfaction levels as “very good” or “good” was 43.8% (*n* = 14) and 25% (*n* = 8), respectively, the number of patients that stated their satisfaction level as “poor” was 5 (15.6%). The ratio of the patients in the group PN that stated their satisfaction levels as “very good” or “good” was 31.3% (*n* = 10) and 59.4% (*n* = 27), and the ratio of those with “poor” satisfaction level was 6.3% (*n* = 2) (Figure 5). As regards patient satisfaction, patients in Group PN stated greater satisfaction rates as compared to the patients in Group P (*P* < 0.05) (*χ* ^2^ = 9.524; *P* = 0.023) (Figure 5).

## 5. Discussion

RIVA is a safe and easy regional anesthesia method used for the upper extremity procedures. The most important complication is the sudden entry of the local anesthetic into the systemic circulation as a result of the accidental opening of the tourniquet or following the deflation of the tourniquet and consequent toxic reactions [12, 13].

Premedication reduces the reaction of the patients to the pneumatic tourniquet [14, 15]. Administration of premedication is recommended in RIVA to prevent the convulsions that might be caused by local anesthetic agents and increase the threshold value of the toxic findings [2, 14]. In our study, 0.07 mg/kg IM midazolam was administered to all the cases.

Since the procedures planned for the patients were of short duration, we also preferred the use of prilocaine 2%. In the studies carried on RIVA, efforts have been exerted to improve the quality of anesthesia by adding several adjuvant substances to local anesthetics [5–7, 16, 17]. We also added NaHCO_3_ to prilocaine in our study and planned to investigate the effects on the tourniquet pain and the onset and termination times of motor and sensory blocks.

PKa values of local anesthetics are close to the pH of the plasma and extracellular fluid 7.4 and range between 7.6 and 8.9. Local anesthetics are found in two forms: the fat-soluble nonionized free base and the water-soluble ionized form. This comparative ratio is dependent on the PKa value of the drug and pH of the tissue. PKa values of local anesthetics are constant. Increasing the free base ratio is possible through increasing the pH of the solution. Penetration to the nerve and onset time of the block is increased this way [18]. Adding alkalinized substances to the local anesthetic solutions increases the local anesthetic effect by increasing the proportion of the nonionized form and making penetration to the body of the nerve easier [2, 11].

Use of tourniquet in upper and lower extremity procedures is defined as the tourniquet discomfort and causes distress. Tourniquet pain is poorly localized and starts right after the inflation of the tourniquet cuff emerging as progressive feelings of burning sensation or ache; it disappears completely with the deflation of the tourniquet. Tourniquet pain can be felt despite the adequate anesthesia during the procedure; despite role of *α*-*δ* fibers and nonmyelinated C-fibers, it has not been fully explained yet [19–22]. Chabel et al. [23] showed the spontaneous afferent neuronal activity appearing following the inflation of the tourniquet in their neurophysiologic studies on rats. It has been reported that these activities do not respond to mechanical stimulations distal to the tourniquet, local anesthetics, or cold block application and disappear only with the blocking of the proximal of the tourniquet or with the deflation of the tourniquet.

In their study which they carried out by adding NaHCO_3_ to prilocaine, Armstrong and colleagues [24] found that alkalization speeded up the onset of sensory and motor blocks and slowed down the termination of the sensory block. In our study, we found that it was not effective on the onset and termination times of the sensory block and also on the onset time of the motor block; however, it elongated the termination of the motor block. After the infusion of sodium bicarbonate into the venous system of the patient which has already been blocked by using tourniquet inflation, the resulting chemical reaction in the blood with water will produce considerable amount of CO_2_. This gas can penetrate easily into the interstitial space of the limb and, as a result, a profound acidosis in that space ensues. This acidosis can tell us why the onset time of both motor and sensory block did not differ between groups. After the release of tourniquet and flushing of fresh blood into the ischemic hand, the extra CO_2_ has been washed out and the interstitial space become again more alkaloid, so the level of unionized local anesthetics increased and probably its effects became more profound and prolonged. Also we used the NaHCO_3_ in a very low dose (2 mec), which we do not expect to cause a severe acidosis. This may be the reason behind significant difference between two groups in terms of the time to termination of motor block. In a study carried out by Solak and colleagues [25], it was found that adding NaHCO_3_ to prilocaine improved the quality of anesthesia. In our study, very good and good patient satisfaction levels were evaluated as 97% in Group PN, which was the group in which NaHCO_3_ was added to prilocaine, while the same was 68.8% in Group P (*P* < 0.05). Capogna and colleagues [26] investigated alkalization in epidural, sciatic, and femoral and brachial plexuses and found that alkalization in blocks made using various local anesthetics speeded up the onset of the sensory and motor blocks.

In separate studies of Armstrong et al. and Solak et al, [24, 25] they reported that the addition of sodium bicarbonate to prilocaine speeded up the onset of the sensory block time. However, the onset time of the sensory block was 8.88 ± 5.90 minutes in Group P and 8.44 ± 5.95 minutes in Group PN, which were different from the other studies. This difference can be related to the amount of the solution used. The amount of solution was 40 mL in the RIVA technique used in various studies. Solak et al. [25] reported that 30 mL solution was used and no difference was found from other studies as regards the onset of the sensory block. Taking this into consideration, we used 20 mL solution in our study.

In a study, no significant differences were found between the onset and termination times of motor block [17]. The motor block onset times were similar in Groups P and PN with 14.46 ± 8.32 minutes and 14.59 ± 7.92 minutes in our report, respectively. While the termination time of the motor block in Group P was 46.79 ± 7.20 minutes, the same in group PN was 55.09 ± 19.04 minutes, and this result was evaluated as statistically significant (*P* < 0.05). Addition of sodium bicarbonate to prilocaine had elongated the motor block period significantly.

In RIVA, anesthetic effect disappears shortly after opening the tourniquet and analgesic drugs are needed in the postoperative period. When the use of additional drugs was evaluated, it was seen that no additional drugs were used in 14 patients in Group P and 9 patients in Group PN. Fentanyl was used in 10 patients in Group P and only in 5 patients in Group PN, although statistically similar rates of usage was concluded in both groups. This result was evaluated as statistically significant (*P* < 0.05). However, dormicum use was found to be significantly different in Group PN as compared to Group P (*P* < 0.05).

Brown and colleagues [14] reported that they observed no cardiovascular system changes except for medium-level bradycardia after RIVA application using lidocaine, prilocaine, and bupivacaine among 906 patients throughout 20 years. No intraoperative or postoperative pulse changes were observed in our study.

No statistically significant differences were found between the two groups in the intraoperative and postoperative OAP, HR, and SpO_2_ values in several studies [23, 27]. In our study also, no changes were observed in OAP and HR values that would require treatment between the groups. When the SpO_2_ values in groups were compared, no statistically significant changes were found in the two groups as regards the SpO_2_ values in preoperative and postoperative periods.

Patient satisfaction was evaluated as very good, good, medium, and poor. While the percentage of patients evaluated as very good and good in Group PN was 97%, the same was found as 68.8% in Group P. The patient satisfaction was found significantly higher in Group PN as compared to Group P (*P* < 0.05).

## 6. Conclusion

We observed that alkalization of prilocaine reduced the tourniquet pain as compared to prilocaine, elongated the termination period of the motor block, reduced the tourniquet pain more as compared to prilocaine, elongated the termination period, increased the patient satisfaction, and reduced the use of intraoperative fentanyl use.

When we compared the effects of prilocaine and alkalinized prilocaine, we observed that alkalization did not have any effects on the onset and termination times of sensory block and onset time of the motor block, and also there were no significant differences between the two groups as regards the onset time of tourniquet and postoperative pains and hemodynamic parameters and adverse effects.

## Figures and Tables

**Table 1:** Demographic characteristics, ASA scores, and tourniquet times of the patients according to groups.

Demographic characteristics	Group P	Group PN	Test statistics	
	*n* (%)	*n* (%)	*χ* ^2^	*P*

Gender				
Male	9 (28.1)	7 (21.9)	0.333	0.564
Female	23 (71.9)	25 (78.1)		

	Median (IQR)	Median (IQR)	*Z*	*P*

Age (years)	50.0 (16.0)	52.0 (16.8)	1.365	0.172
Body Weight (kg)	70.0 (18.8)	74.5 (10.0)	0.813	0.416
Tourniquet Time (minutes)	41.0 (8.0)	40.0 (11.5)	0.525	0.600

	*n* (%)	*n* (%)	*χ* ^2^	*P*

ASA Score				
Normal, healthy	11 (34.4)	11 (34.4)	2.171	0.338
Mild systemic disease	17 (53.1)	20 (62.5)		
Severe systemic disease	4 (12.5)	1 (3.1)		

**Table 2:** Surgical procedures used.

Procedure types	Group P		Group PN	
	*N*	%	*n*	%
Ganglion cyst excision	5	15.6	9	28.1
Excision of mass lesion from the finger	7	21.9	6	18.8
Carpal tunnel syndrome	12	37.5	10	31.3
Finger fracture	0	0.0	1	3.1
Trigger finger	8	25.0	6	18.8

Total	32	100	32	100

**Table 3:** The onset and termination times of sensory and motor blocks in Groups P and PN.

	Group P	Group PN	P-PN comparison	
	Mean SD	Mean SD	*T*	*P*
**Onset of sensory block (min)**	8.88 ± 5.90	8.44 ± 5.95	**0.296**	**0.769**
Termination of sensory block (min)	102.13 ± 35.44	99.03 ± 25.45	0.401	0.690
**Onset of motor block (min)**	14.46 ± 8.32	14.59 ± 7.92	**0.062**	**0.951**
Termination of motor block (min)	46.79 ± 7.20	55.09 ± 19.04	2.175	**0.034**

*P < 0.05.

**Table 4:** Verbal Analog Scale (VAS).

0–2	No pain
2–4	Mild pain
4–6	Medium-level pain
6–8	Severe pain
8–10	Very severe pain

**Table 5:** Use of additional drugs according to groups.

	Group P		Group PN	
	*N*	%	*n*	%
Additional drug use				
No	14	43.8	9	28.1
Yes	18	56.2	23	71.9
**Fentanyl**	**2∗**	**6.2**	**3∗**	**9.4**
Dormicum	8	25.0	18∗∗	56.2
**Fentanyl + Dormicum**	**8∗**	**25.0**	**2∗**	**6.3**

*While fentanyl was administered to 10 patients in Group P, fentanyl was used in 5 patients in the Group PN.

**P < 0.05.

**Table 6:** Distribution of Bromage Scale in Groups P and PN.

Bromage Scale	Group P		Group PN		Total	
	*N*	%	*n*	%	*n*	%
Full motion	3	9.7	1	3.1	4	**6.3**
Wrist	3	9.7	2	6.3	5	**7.9**
Fingers	6	19.4	5	15.6	11	**17.5**
Motionless from the elbow	19	61.3	24	75.0	43	**68.3**

TOTAL	**31**	**100.0**	**32**	**100.0**	**63**	**100.0**
